# Deltamethrin-induced oxidative stress and biochemical changes in tissues and blood of catfish (*Clarias gariepinus*): antioxidant defense and role of alpha-tocopherol

**DOI:** 10.1186/1746-6148-8-45

**Published:** 2012-04-26

**Authors:** Kamal A Amin, Khalid S Hashem

**Affiliations:** 1 Faculty of Veterinary Medicine, Department of Biochemistry, Beni Suef University, 62511, Beni Suef, Egypt

**Keywords:** Deltamethrin, *Clarias gariepinus*, Egypt, Oxidative biomarkers, Catalase, Malondialdhyde, α-tocopherol

## Abstract

**Background:**

The pyrethroid class of insecticides, including deltamethrin, is being used as substitutes for organochlorines and organophosphates in pest-control programs because of their low environmental persistence and toxicity. This study was aimed to investigate the impact of commonly used pesticides (deltamethrin) on the blood and tissue oxidative stress level in catfish (*Clarias gariepinus)*; in addition to the protective effect of α-tocopherol on deltamethrin induced oxidative stress.

Catfish were divided into three groups, 1^st^ control group include 20 fish divided into two tanks each one contain 10 fish, 2^nd^ deltamethrin group, where Fish exposed to deltamethrin in a concentration (0.75 μg/l) and 3^rd^ Vitamin E group, Fish exposed to deltamethrin and vitamin E at a dose of 12 μg/l for successive 4 days.

Serum, liver, kidney and Gills were collected for biochemical assays. Tissue oxidative stress biomarkers malondialdhyde (MDA) and catalase activity in liver, kidney and gills tissues, serum liver enzymes (ALT and AST), serum albumin, total protein, urea and creatinine were analysed.

**Results:**

Our results showed that 48 h. exposure to 0.75 μg/l deltamethrin significantly (*p* < 0.05) increased lipid peroxidation (MDA) in the liver, kidney and gills while catalase activity was significantly decreased in the same tissues. This accompanied by significant increase in serum ALT, AST activity, urea and creatinine and a marked decrease in serum albumin and total proteins.

**Conclusions:**

It could be concluded that deltamethrin is highly toxic to catfish even in very low concentration (0.75 μg/l). Moreover the effect of deltamethrin was pronounced in the liver of catfish in comparison with kidneys and gills. Moreover fish antioxidants and oxidative stress could be used as biomarkers for aquatic pollution, thus helping in the diagnosis of pollution. Adminstration of 12 μg/l α-tocopherol restored the quantified tissue and serum parameters, so supplementation of α-tocopherol consider an effective way to counter the toxicity of deltamethrin in the catfish.

## Background

For centuries, pesticides have been used in agriculture to enhance food production by eradicating unwanted insects and controlling disease vectors. Among common pesticides are organophosphorus compounds which are widely used in agriculture, medicine and industry. The main advantages of pyrethroids that made it successively replacing organophosphrus pesticides are their photostability, high effectiveness even in low concentration, easily disintegration, and low toxicity in birds and mammals [1].

Fish and various other aquatic organisms are extremely susceptible to pyrethroids as the 96 h Lc50 value determined in laboratory tests, generally lies below 10 μg/l. In addition, deltamethrin is based on pyrethroids that have established significantly lower rates of metabolism and removal in fish than those recorded for birds and mammals [2].

Effluents of agricultural and industrial processes contain highly toxic chemicals like pesticides that lead to pollution of aquatic environments including rivers, ponds and lakes. The accumulation and persistence of insecticide and pesticides in the aquatic environment constitute a threat to biological life, as witnessed by the chronic and acute poisoning of fish and other aquatic organisms [3].

Deltamethrin has very good residual activity for outdoor uses and for indoor uses; it has a high toxicity to fish under laboratory environment. However, in field conditions under normal conditions of use, fish may not harmed. Deltamethrin had an impact on aquatic herbivorous insects. This impact led to an increase of algae.

The World Health Organisation reported that roughly 3 million cases of pesticide poisoning occur annually that result in 22 000 deaths worldwide. Many of these chemicals are mutagenic [4] linked to the development of cancers or may lead to development deficits [5].

*Clarias* species are air breathing fishes due to the presence of accessory assistant respiratory organs beside the gills enabling it to survive for long time outside the water, otherwise debilitating hypoxic environments [6]. Catfish *Clarias* is freshwater, belonging to the genus *Clarias*. There are 32 species of catfish belonging to genus *Clarias* are known in Africa. *Clarias lazera* and *Clarias gariepinus* are the most popular members of the inland water fishes found in Egypt.

*Clarias lazera* is known locally as karmout and is commonly found in Lake Nasser and all Nile branches and streams [7]. These species are tolerant to a wide range of water and laboratory conditions and has detritivorous behaviour. This means that the fish can be in contact with xenobiotics from different ways of interacting with algae from stone or sediment. These characteristics make this particular species an interesting model for ecotoxicological and biochemical studies. Moreover Catfish are valuable bio-indicators of contamination because of their large distribution, being open swimmers, capacity to react against ecological pollution and food source for human.

The long-term biological hazards associated with the use of organochloride, organophosphate and carbamate pesticides propelled the introduction of a new generation of pesticides with a lesser degree of persistence. In this direction, the use of pyrethroids as insecticidal and antiparasitic formulations has markedly increased as a viable substitute and account for over 30% of insecticides used globally [8].

Deltamethrin [((s)-a-cyano-3-phenoxybenzyl(R1-R2)-3-(2,2 dibromvinyl)-2,2 dimethylcyclo-propancarboxylate] is one of the most important widely used pyrethroids pesticide and insecticides, since the application of pyrethroid as insecticide and antiparasitary preparations has been accepted on a large scale for agricultural purposes and very markedly increased during last 10–15 years; even though it is already known that this insecticide is highly toxic to fish and various other aquatic organisms [9].

The presence of low deltamethrin concentration in water has sublethal effects such as altered energy metabolism and ionic regulation. It is a synthetic type two of pyrethroids, which has a wide range of application in industrial and agricultural purposes. It is also used as an alternative pesticide in animal health, in vector control, and in public health [10].

Among the most commonly used biomarkers, those related to oxidative stress assume an important position, being frequently used both in environmental monitoring and laboratory assays [11].

Rates or amounts of reactive oxygen species (ROS) production can be increased by the presence of a wide range of natural and man-made xenobiotics [12].

The stimulation of free radical production, induction of lipid peroxidation, and disturbance of the total antioxidant capability of the body are mechanisms of toxicity for most pesticides, including organochlorines and pyrethroids [13]. Consequently, the antioxidant defences are potentially interesting biomarkers to pesticides while enhanced lipid peroxidation, a consequence of oxidative deterioration of membrane lipids, is generally referred to an index of oxidative stress. Moreover, ROS alter protein structure or function and Amino acid side-chains can be irreversibly modified into aldehyde or ketone groups (carbonylation) which can lead to protein aggregation, inactivation or degradation, these changes in protein carbonylation process are a biochemical perturbation resulting from oxidative stress [14].

Few reports have demonstrated the induction of oxidative stress by pyrethroids, such as cypermethrin and fenvalerate [15], while the effect of deltamethrin on catfish have not been investigated. Induction of oxidative stress is one of the main mechanisms of many pesticides action [13]. The damage to membrane lipids, protein and DNA is the endpoint biomarker of oxidative stress-inducing effects of pesticides [16]. Various adaptive and compensatory responses are also induced as a result of exposure to deltamethrin in fish [17].

α-tocopherol, a fat-soluble vitamin, is a major antioxidant, responsible for terminating free radical chain reactions that result from the oxidation of polyunsaturated fatty acids (PUFA) [18]. Therefore, it was suggested an increased amount of α-tocopherol supplementation to inhibit cellular lipid peroxidation of PUFA.

In order to protect the tissue from oxidant injury, antioxidant enzymes are also present in the biological system. Blomhoff [19] reported that when animals are exposed to a dietary oxidative stress, they react with compensatory induction of endogenous antioxidants. Supplementation of dietary components like vitamin E [tocopherols and tocotrienols) and vitamin C, etc. lowered lipid peroxidation, protein oxidation and the incidence of various morbidities or mortalities; induced catalase activity, reduced the oxidative stress in rat.

The contamination of aquatic ecosystem by pesticides has gained increasing attention in recent decades. The acute and chronic exposure and accumulation of these chemicals can result in tissue burdens that produce adverse effects not only in the exposed organisms, but also organisms including human beings; therefore, it seems essential to study detrimental effects of such hazardous pollutants so as to formulate the strategies for safe guarding aquatic organisms.

The present study was designed to assess the effect of two doses of deltamethrin on several biochemical parameters on serum and tissues of catfish (*Clarias gariepinus*) to establish this species as a model for biochemical studies. Also, we aimed to investigate the effects of deltamethrin on some oxidative biomarkers (MDA and catalase activities) and some serum biochemical parameters, alanine transaminase (ALT), aspartate transaminase (AST), total protein, albumin, urea and creatinine. In order to better understand fish response to deltamethrin exposure and try to use α-tocopherol in order to restore the alteration of the changes in the oxidative and biochemical parameters which are dysregulated by deltamethrin.

## Materials and methods

### Material

#### Fish rearing and management

A total of 40 immature channel catfish of both sexes were collected from different localities of *El-Ebrahemia* channel average weighted 100 ± 5 g were reared in water aquaria for one week for acclimation before the beginning of the experiment [15]. The water temperature about 22°C-23°C and pH level kept at 7.6 ± 0.4. Water was supplied with oxygen by air pump. The photoperiod was a 12-h light to 12-h dark cycle and the fish were fed on commercial ration at ratio of 3% of body weight/day.

#### Chemicals

The following chemicals were used: Deltamethrin were purchased as a commercial product (Butox) 50 mg/ml. α-tocopherol were purchased as a solution of 10% from the El Naser Pharmaceutical Company (*Cairo*-*Egypt*).

### Methods

This experimental research was approved by the Committee of Scientific Ethics at Beni Suef University, faculty of veterinary medicine , Biochemistry department and consistent with its guidelines.

#### Experimental design and Fish grouping

Catfish were divided into three groups, as follows:

*Control group*: 20 fish divided into two tanks each one contain 10 fish. Fish were kept in 10 liters of de-chlorinated water. Water was changed once daily.

*Deltamethrin group:* Fish exposed to deltamethrin in a concentration of 0.75 μg/l [16,17,20] for 48 hours as follow, 10 fish were put in 10 liters of de-chlorinated water tank contains deltamethrin in concentration of 0.75 μg/l. To keep a constant concentration of deltamethrin, water was sucked daily and the tank was supplied by water with deltamethrin in the same concentration daily for 2 days.

*Vitamin E group:* Fish exposed to deltamethrin in a concentration (0.75 μg/l) for 48 hours as mentioned before. In the third day, fish were supplied by 10 liters of water contain vitamin E at a dose of 12 μg/l for successive 4 days. To keep a constant concentration of deltamethrin, water was sucked daily and the tank was supplied by water with vitamin E in the same concentration daily for 4 days.

#### Sampling and tissue preparation

##### Serum samples

At the end of the experiment (1 week post administration of chemicals) fish were caught by a net and blood collected from the caudal peduncle then transferred to clean dry centrifuge tubes then left for 2 h for clotting and centrifuged at 3000 rpm for 15 min, followed by serum separation and storage in deep freeze till biochemical analysis.

##### Tissue samples

Liver; kidneys and gills of scarified fish were rapidly removed, cleaned from any extraneous materials and immediately perfused with saline. The tissues were homogenised in phosphate buffer (0.1 M pH 7.4).the homogenate was filtered and centrifuged at 1 500 rpm for 20 min .The supernatant was stored in deep freeze until biochemical analysis.

#### Biochemical analysis of serum and tissue

Serum ALT and AST were measured colourimetrically according to method described by Reitman *et al.*[21] using kits purchased from Diamond Diagnostic (Cairo, Egypt). Serum total protein and albumin were measured according to Lubran [22] and Doumas *et al.*[23], respectively. Also measurements of serum urea [24] and serum creatinine [25] were considered using spectrophotometer (Hitachi 2000).

Lipid peroxide was measured in tissues according to method of Satoh [26] that based on the ability of thiobarbituric acid (TBA) to reacts with malondialdhyde (MDA) in acidic medium at temperature of 95°C for 30 min to form TBA reactive product the absorbance of the resultant pink product can be measured at 534 nm. Tissue catalase activities were measured according to Aebi *et al.*[27].

### Statistical analysis

The obtained data were statistically analyzed by ANOVA test using PC-STAT Statistical programs and data were reported as mean ± SEM.

## Results

The results are summarised in tables and figure as follows:

Table 1: demonstrates that deltamethrin (0.75 μg/l) significantly increased the activities of AST and ALT, while significantly decreased the level of serum total protein and albumin compared to control group. In addition exposure to α-tocopherol significantly improved ALT and AST activities, while significantly increase concentration of serum total protein and albumin in comparison with deltamethrin group. α-tocopherol alleviated the toxicity and negative effect of deltamethrin on the activities of the measured enzymes.

**Table 1:** Effects of deltamethrin (0.75 μg/l) and α-tocopherol on serum liver enzymes activities in catfish

**Groups**	**ALT (IU/l)**	**AST (IU/l)**	**T. proteins (g/dl)**	**Albumin (g/dl)**
Controls	31.97 ± 1.82^(a)^	20.99 ± 3.43^(a)^	4.55 ± 0.13^(a)^	1.83 ± 0.05^(a)^
Deltamethrin (0.75 μg/l)	61.58 ± 5.66^(a)^	55.67 ± 7.54^(b)^	3.65 ± 0.2^(b)^	1.46 ± 0.08^(b)^
α-tocopherol (12 μg/l)	26.80 ± 3.40^(a)^	11.97 ± 1.22^(a)^	4.66 ± 0.39^(a)^	1.91 ± 0.14^(a)^

ALT, alanine transaminase activitiy.

AST, aspartate transaminase activitiy.

Values are expressed as means ± SEM.

Means with different supercripts are significantly different (*p* < 0.05), while means with the same superscripts indicate non-significant changes.

Table 2: shows that MDA level (LPO) was significantly increased, in liver, kidney and gills of fish treated with deltamethrin in the same dose. Exposure to α-tocopherol caused significant reduction in the elevated MDA and maintain to its normal values in liver, kidney and gills MDA in comparison with deltamethrin group and control.

**Table 2:** Effects of deltamethrin and α-tocopherol on liver, Kideny and gill malondialdhyde levels

**Groups**	**Liver MDA (nmol/g tissue)**	**Kidney MDA (nmol/g tissue)**	**Gill MDA (nmol/g tissue)**
Controls	21.49 ± 0.38^(a)^	12.91 ± 0.23^(a)^	8.58 ± 0.15^(a)^
Deltamethrin (0.75 μg/l)	33.10 ± 1.79^(b)^	19.87 ± 1.07^(b)^	13.24 ± 0.71^(b)^
α-tocopherol (12 μg/l)	21.59 ± 0.77^(a)^	12.93 ± 0.46^(a)^	8.64 ± 0.31^(a)^

MDA, malondialdhyde.

Values are expressed as means ± SEM.

Means with different superscripts are significantly different (*p* < 0.05), while means with the same superscripts indicate non-significant changes.

Table 3: illustrates that catalase activities were significantly increased, in liver, kidney and gills in fish treated with deltamethrin when compared to control group. Exposure to α-tocopherol caused significant elevated the dysregulation of catalase activity in Liver, kidney and gills in comparison with deltamethrin group.

**Table 3:** Effects of deltamethrin and α-tocopherol on Liver, kidney and gill catalase activity in catfish

**Groups**	**Liver catalase (u/g. tissue)**	**Kidney catalase (u/g tissue)**	**Gill catalase (u/g tissue)**
Controls	0.26 ± 0.012^(a)^	0.35 ± 0.01^(a)^	0.33 ± 0.009^(a)^
Deltamethrin (0.75 μg/l)	0.16 ± 0.019^(b)^	0.29 ± 0.011^(b)^	0.25 ± 0.012^(b)^
α-tocopherol (12 μg/l)	0.27 ± 0.021^(a)^	0.35 ± 0.012^(a)^	0.34 ± 0.014^(a)^

(u/g.) Unit/gm tissue.

Values are expressed as means ± SEM (standard error mean).

Means with different superscripts are significantly different (*p* < 0.05), while means with the same superscripts indicate non-significant changes.

**Figure 1:**
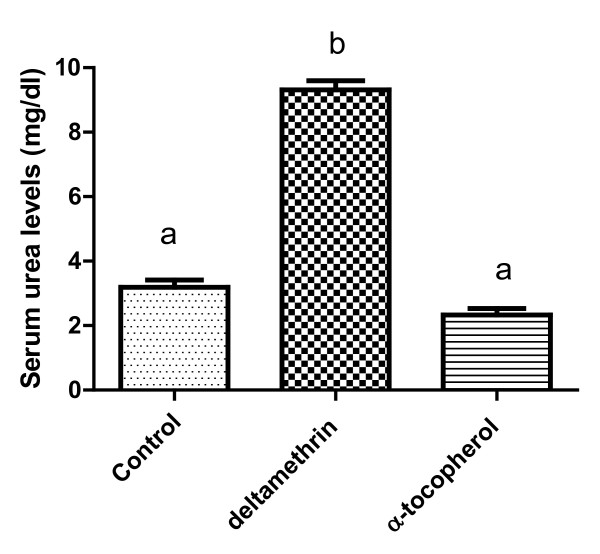
**Effects of deltamethrin and α-tocopherol on serum urea levels in catfish.** Serum urea level increased significantly in catfish giving deltamethrin compared with values in the control group. Exposure to α-tocopherol decreased and maintained these levels to the normal values.

**Figure 2:**
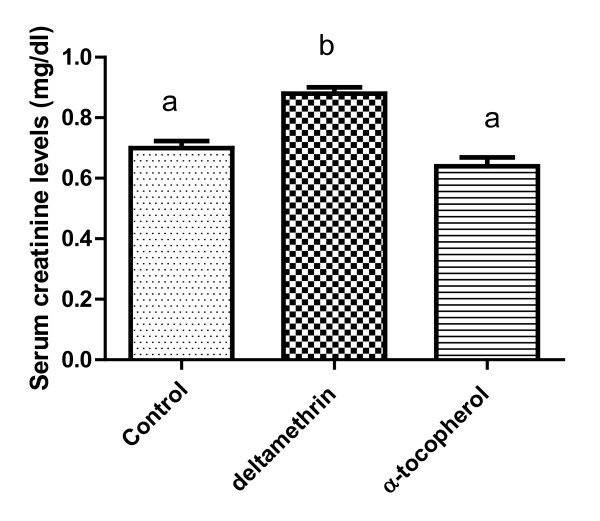
**Effects of deltamethrin and α-tocopherol on serum creatinine levels in catfish.** Serum creatinine level elevated significantly in catfish administered with deltamethrin, compared with control group, while exposure to α-tocopherol ameliorated this effect compared with deltamethrin group.

Figures 1 and 2 show that serum urea and creatinine was significantly increased in catfish treated with deltamethrin, while exposure to α-tocopherol decreased and maintained these levels to the normal values.

## Discussion

### Effect of deltamethrin on liver function and its modulation by α-tocopherol

The evaluation of serum AST and ALT activities, T. proteins, albumin and hepatic MDA level and catalase activity bears significance with respect to the evaluation of the effects of pesticides on the liver, therefore their toxicity [28].

The current results showed significant increase in plasma ALT and AST activities (Table 1). The increase in activity of these enzymes in plasma is indicative for liver damage and thus causes alteration in liver function [29,30]. In addition, El-Demerdash *et al.*[15] found that cell damage exhibited good correlation with the enzyme leakage. Hence, cellular damage caused by toxic substances is frequently accompanied by increasing cell membrane permeability.

The decrement of plasma total protein, albumin (Table 1) comes in agree with Yousef *et al.*[29,30] and El-Demerdash *et al.*[15]. This decrease in plasma protein could be attributed to changes in protein and free amino acids metabolism and their synthesis in liver. Also the observed decreases in plasma proteins could be attributed to the damaging effect of deltamethrin on liver c ells as confirmed by increasing in the activities of ALT and AST.

### Effect of deltamethrin on renal function tests and its modulation by α-tocopherol

Blood urea levels were significantly elevated in deltamethrin exposed group in comparison with control group (Figure 1), this elevation may be due to correlation between urea and increased protein catabolism or from more efficient conversion of ammonia to urea as a result of increased synthesis of enzyme involved in urea production. The high levels of blood urea and creatinine (Figure 2) result either from increased breakdown of tissue or dietary or impaired excretion or increased synthesis or decreased urinary clearance by the kidney or decreased degradation of these compounds [31]. The present data suggest that polluted fish adept glomerular dysfunction rather than tubular insufficiency as blood levels of urea and creatinine depend largely on glomerular function.

In consistent with these explanations of decreased total protein level with deltamethrin administration, urea is the end product of protein catabolism in mammals but in fish ammonia is the end products of protein, So the marked increase in blood urea nitrogen could be attributed to impaired excretion of urea through kidney and this explanation is supported by increasing blood creatinine level which is more sensitive and specific indicator of impaired kidney function [32].

### Effect of deltamethrin on liver, kidneys and gills oxidative stress and its modulation by α-tocopherol

Fish gills, kidneys and liver are critical organs for their respiratory, osmoregulatory and excretory functions. Respiratory distress is one of the early symptoms of pesticide poisoning. A high rate of absorption of deltamethrin through gills also makes fish a vulnerable target of its toxicity [33].

Some studies indicated that exposure to lethal concentrations of deltamethrin caused destructive effect in the gill, kidney and liver tissues of *C. carpio*. The catfish gill is a multipurpose organ that plays role in water gas exchange, osmotic and ionic regulation, acid–base balance and elimination of nitrogenous wastes so, Gill and kidney tissues alterations may result in severe functional problems, ultimately leading to the death of fish.

A single 48 h. exposure to deltamethrin-induced oxidative stress in all the tissues, as depicted by elevated levels of MDA in tissues. Deltamethrin is known to alter cell metabolism in various ways, with a potential genotoxic risk, including DNA damage and micronucleus induction in freshwater fish [34].

The present findings implicate a role of oxidative stress and free radical formation in these effects. Studies in rodents have demonstrated that absorbed deltamethrin is readily metabolised and excreted; elimination is achieved within 2–4 days. The major metabolic reactions ascribed to deltamethrin metabolism are oxidations mediated by the microsomal monooxygenase system.

The degradation pathways in cows, poultry, and fish are almost similar to those in rodents. However, comparative *in vivo* and *in vitro* metabolic studies have shown that fish have a lower capacity to metabolize and eliminate pyrethroid insecticides [35]. This is reflected in the present investigation, where deltamethrin induced peroxidative damage in all the tissues and the gills in particular during a short-term subacute exposure regimen. The extent of MDA is determined by the balance between the production of oxidants and the removal and scavenging of those oxidants by antioxidants [36].

Gills are the primary sites for the absorption of deltamethrin. It is, therefore, obvious that we noted a high level of MDA coupled with depletion of antioxidant enzymes in the gills. Lipid peroxidation has been extensively used as a biomarker of oxidative stress [37].

MDA are produced by LPO and considered as indicators of oxidative stress, which results from the free radical damage to membrane components of cells.

Fish exposure to deltamethrin-induced oxidative stress in all tissues, as depicted by elevated levels of MDA in tissues [Table 2). Deltamethrin is known to alter cell metabolism in various ways, with potential genotoxic risk, including DNA damage and micronucleus induction. Comparative in vivo and in vitro metabolic studies have shown that fish have a lower capacity to metabolise and eliminate pyrethroid insecticides [35].

In the present study exposure of catfish to 0.75 μg/l concentration of deltamethrin (Table 2) shows a marked increase in MDA in the liver, kidney and gill [38,39]. This marked increase could be attributed to the ability of deltamethrin for free radical formation.

Catalase activity was significantly reduced in the deltamethrin group when compared to control one (Table 3) and this in agreement with Pandey *et al.*[38]. This significant reduction could be attributed to the influx of super oxide radicals, which have been reported to decrease CAT activity.

The current results showed that deltamethrin is highly toxic to fish even in low concentration [0.75 μg/l) while sublethal concentration of deltamethrin was [1.5 μg/l) as reported by Svobodova *et al.*[40]. This highly toxic effect of deltamethrin in fish is due to the high rate of absorption of deltamethrin through the gills and lack of fish to the enzymes responsible for metabolism and detoxification of deltamethrin [17].

The decreased CAT activities, total proteins and albumin, while increased MDA level in liver as well as increased serum AST and ALT activities suggest that deltamethrin produces hepatic dysfunction. The pathogenesis may be through free radical formation where deltamethrin undergoes metabolism in the liver via hydrolytic ester cleavage and oxidative pathways by the cytochrome P450 microsomal enzyme system which probably decreased the P450 contents in liver that may causes in oxidative stress producing depletion of CAT activity and increased the level of MDA leading to hepatic degeneration and necrosis [41].

α-tocopherol considered the principal antioxidant defence against lipid peroxidation in cell membranes of mammals. The most important role of α-tocopherol in tissues seems to be the protection of membrane PUFA against the effects of oxygen radicals It inhibits peroxidation of membrane lipids by scavenging lipid peroxyl radicals, and is converted into a tocopheroxyl radical as a consequence [18]. α-tocopherol selectively blocks the pyrethroid-modified sodium channel in a dose-dependent manner without affecting normal sodium channels. Moreover α-tocopherol maintained the activities of membrane-bound enzymes at near normal values, and thus preserving mitochondrial membrane integrity and protected some enzyme activities from oxidation by free radicals [42].

Vitamin E dietary supplementation decreased the susceptibility to lipid peroxidation in rat tissues and peroxidation of rat liver microsomes induced by Fe/ascorbate [18]. The malondialdehyde production in tissue homogenates is used as an estimate of lipid peroxidation.

The protective effect of α-tocopherol against deltamethrin that induced elevation of serum and tissues MDA, observed in this study, was harmony with its antioxidant activity and might also be relevant to its prophylactic and therapeutic effect against environmental pollutants.

ROS have been implicated in the toxicology of pyrethroids, so the protective effect of α-tocopherol, observed in our study, could be important for protecting the different tissues against the oxidative injury following the use of deltamethrin. So fish fed a low level of dietary α-tocopherol failed to counter the stress of deltamethrin pollution.

The results in Table 2 showed that lipid peroxidation in liver was higher than MDA in kidneys and gills also catalase activity were reduced in liver in comparison with kidneys and gills respectively this is due to the fact, that liver is the main organ for metabolism and detoxification of deltamethrin [16,43], demonstrating that the toxicity of deltamethrin was pronounced in the liver of catfish in compare with kidneys and gills this indicate that liver is the main affected organ by pollution and the most sensitive for oxidative stressors.

In general, antioxidant enzyme activity in kidney and gills was less evident than in liver. Oxidative stress indices (levels of MDA) were significantly higher in liver, despite a trend to increased values were manifested in the remaining tissues. In short, deltamethrin-induced stress responses in different tissues were reflected in the oxidative stress indices and liver function parameters. Those parameters may use as biomarkers for monitoring residual pharmaceuticals in aquatic environment, However more detailed experiments in laboratory need to be performed in the future.

Alterations in serum urea, creatinine (Figures 1 and 2), renal MDA and catalase are encountered primarily in dysfunction and injure of the kidneys, while gill MDA and catalase are markers of gill function.

The hepatic antioxidant enzyme (CAT) activity was inhibited significantly at concentrations (0.75 μg/l) of deltamethrin. Meanwhile, the antioxidant enzyme activity was significantly inhibited in kidney and gills of fish. Moreover there was significant higher MDA level in hepatic, renal and Gill tissues in deltamethrin group compared with control. In short, concentrations of (0.75 μg/l) deltamethrin could induce obvious impacts on different organs in fish, and could affect seriously the health status of fish.

This biochemical information will be helpful in assessing the impact of deltamethrin toxicity to freshwater fish as well as in developing a sensitive biomarker of aquatic pollution caused by the excessive release of deltamethrin into the freshwater.

Alterations of lipid peroxidation and catalase activity after deltamethrin exposure suggesting the use of these antioxidants as a potential biomarker of toxicity associated with contaminant exposure in freshwater catfish.

Application of specific concentration of heavily used deltamethrin with the Egyptian catfish that grow in many water areas, using serum and tissues biochemical makers for monitoring pollution and treatment with natural vitamin E as antioxidant through water and making sure it taken by catfish not forming a film, provides novelty to our study.

## Conclusions

It could be concluded that, deltamethrin is highly toxic to catfish even in very low concentration (0.75 μg/l) in spite of major researches where they reported that deltamethrin is safe for living organisms at sublethal concentration (1.5 μg/l).

Moreover our results recommended that oxidative stress may, in part, be contributing to deltamethrin-induced hepatic, renal and gill damage. It may provide an indication of aquatic pollution load of deltamethrin in the affected catfish population, so help in the diagnosis of the pollution. Also our results indicated that α-tocopherol could modulate and diminish the adverse effects of deltamethrin on most of biochemical parameters, lipid peroxidation and enzymatic activities if used in low concentration (12 μg/l). Also, deltamethrin produced oxidative stress in fish gill more than liver and kidney both at catalase activity and MDA levels.

## Competing interests

The authors declare that they have no competing interests.

## Authors’ contributions

KA and KH carried out experimental work; biochemical and statistical analysis, interpretation and discussion of the results wrote the paper, related to their part of the work. All authors perform drafting, read and approved the final manuscript.
